# Simultaneous Determination of 20 Sulfonamide Residues in Pork by Filter-Based Solid-Phase Extraction Cleanup and UHPLC–MS/MS

**DOI:** 10.3390/foods15193465

**Published:** 2026-09-28

**Authors:** Shuang Chen, Shanggui Deng, Yong Zhou, Bin Zhang, Pengxiang Yuan

**Affiliations:** 1 Food Processing and Safety, School of Food and Pharmacy, Zhejiang Ocean University, Zhoushan 316022, China; 2 Zhoushan Institute for Food and Drug Control, Zhoushan 316021, China

**Keywords:** pork, FaVEx, one-step purification column, UHPLC–MS/MS, residue analysis, method validation

## Abstract

To enable rapid and accurate determination of sulfonamide residues in animal-derived foods, a method was developed for the simultaneous analysis of 20 sulfonamides by ultra-high-performance liquid chromatography–tandem mass spectrometry (UHPLC–MS/MS) combined with a FaVEx one-step cleanup column, using pork as a representative matrix. After extraction, samples were rapidly purified with a FaVEx-NM column and analysed in multiple reaction monitoring mode. Matrix-matched calibration was employed to compensate for matrix effects. The method showed good linearity for all analytes within their respective concentration ranges, with correlation coefficients (R^2^) of 0.9922–0.9998. The limits of detection and quantification were 0.05–0.74 μg/kg and 0.09–1.48 μg/kg, respectively, indicating high sensitivity. At spiking levels of 1, 3, and 5 μg/kg, mean recoveries ranged from 70.95% to 112.20%, with relative standard deviations below 11%, demonstrating satisfactory accuracy and precision. The proposed method is simple, efficient, sensitive, and reproducible, and is suitable for rapid screening and quantitative determination of multiple sulfonamide residues in pork, providing reliable technical support for residue monitoring in animal-derived foods.

## 1. Introduction

Animal-derived foods are important sources of high-quality protein and lipids [1], and their safety is closely related to public health and international trade. Veterinary drugs and antimicrobials are widely used in livestock, poultry, and aquaculture to prevent and treat diseases [2], thereby supporting animal health and production efficiency; however, their residues have become a major concern in food safety regulation [3]. Sulphonamides (SAs), synthetic antimicrobials derived from p-aminobenzenesulphonamide [4], are extensively used for bacterial disease control because of their broad antibacterial spectrum, low cost, and chemical stability [5]. Nevertheless, overdosing, improper combined medication, and poor compliance with withdrawal periods remain common in animal production [6], resulting in SA accumulation in edible tissues and products, including muscle, liver, kidney, milk, and eggs [7]. Long-term dietary exposure to SA residues may cause allergic reactions, haematopoietic toxicity, acute or chronic adverse effects, disturbance of the intestinal microbiota, and the development and spread of antimicrobial resistance; some SAs also pose potential teratogenic, mutagenic, and carcinogenic risks [8]. Accordingly, international regulatory authorities and many countries and regions have established strict maximum residue limits (MRLs) for veterinary drug residues in animal-derived foods, with European Union regulations specifying limits for pharmacologically active substances in foodstuffs of animal origin [9].

Current methods for SA residue analysis include immunoassays, capillary electrophoresis, high-performance liquid chromatography (HPLC) [10], and ultra-high-performance liquid chromatography–tandem mass spectrometry (UPLC–MS/MS) [11]. Immunoassays are rapid and simple, making them suitable for preliminary screening, but their quantitative accuracy and confirmatory capability are limited [12,13]. In contrast, UPLC–MS/MS provides efficient separation, high sensitivity, and reliable structural confirmation [14], and has become a key technique for multi-residue analysis of veterinary drugs in animal-derived foods and aquatic products [15,16]. LC–MS/MS and UPLC–MS/MS have been successfully applied to the determination of antibiotics and SAs in complex matrices such as fish, shrimp, livestock, and poultry products, showing high sensitivity, selectivity, and multi-analyte capability [17,18]. However, animal-derived foods contain complex endogenous components, including proteins, lipids, and phospholipids [19,20], which may be co-extracted with target analytes and cause ion suppression or enhancement during electrospray ionisation, compromising analytical accuracy and reproducibility [21]. Therefore, simple, efficient, and matrix-compatible sample preparation is essential for accurate trace-level determination of SAs.

Solid-phase extraction (SPE) is widely used for veterinary drug residue analysis due to its effective clean-up and enrichment performance [22]. Online SPE coupled with UHPLC–MS/MS has improved clean-up efficiency and throughput for SA determination in aquatic samples [23], while eco-friendly SPE-based UPLC–MS/MS methods have been applied to multi-antibiotic determination in fish muscle and dietary exposure assessment [24]. Nevertheless, conventional SPE often involves complex procedures, long processing times, and high organic solvent consumption, limiting its use in high-throughput analysis [25]. Recently, QuEChERS, dispersive SPE, magnetic adsorbents, and modified clean-up techniques have been increasingly used for veterinary drug residue analysis in complex animal-derived foods and aquatic products, improving purification efficiency, reducing matrix effects, and meeting high-throughput requirements [26,27]. Filter-based clean-up columns combine adsorption purification and rapid filtration, enabling efficient matrix interference removal while simplifying sample preparation. Their operational simplicity, rapid processing, and compatibility with high-throughput analysis make them promising for residue analysis in complex food matrices.

In this study, 20 commonly used SAs in animal-derived foods were selected as target analytes, and pork was used as a representative matrix. A rapid, sensitive, and accurate UHPLC–MS/MS method combined with a filter-based clean-up column was developed and optimised for simultaneous SA determination. The method was validated in terms of linearity, limits of detection and quantification, matrix effects, precision, and recovery, providing a practical analytical approach for routine monitoring, risk assessment, and quality control of SA residues in animal-derived foods.

## 2. Materials and Methods

### 2.1. Materials and Reagents

Acetonitrile, methanol (chromatographic grade): Sinopharm Chemical Reagents Co., Ltd. (Shanghai, China); glacial acetic acid (ultra-pure grade), Shanghai McLean Biochemical Technology Co., Ltd. (Shanghai, China); formic acid (mass spectrometry grade), Sinopharm Chemical Reagents Co., Ltd. (Shanghai, China); Acquity UPLC BEHC18 (100 mm × 2.1 mm × 1.7 μm): Waters Corporation, Milford, MA, USA; FaVEx-NM solid-phase extraction column: Green Giant Research Co., Ltd., Hsinchu, Taiwan; 0.22 μm syringe filter: Tianjin Jinteng Laboratory Equipment Co., Ltd. (Tianjin, China). Test samples: Randomly sampled by the Zhoushan Institute for Food and Drug Control (Beijing, China).

The reference standards used included 20 sulphonamide drugs, namely: sulfapyridine (SPY), sulfadiazine (SDZ), sulfathiazole (STZ), sulfamerazine (SMR), sulfamethoxypyridazine (SMP), sulfameter (SME), sulfamethizole (SMT), sulfadoxine (SDX), sulfamonomethoxine (SMM), sulfabenzamide (SBZ), sulfadimethoxine (SDM), sulfaquinoxaline (SQX), sulfaphenazole (SPZ), sulfacetamide (SAA), sulfachloropyridazine (SCP), sulfamethoxazole (SMX), sulfaguanidine (SGD), sulfisomidine (SIM), sulfamethazine/sulfadimidine (SMZ) and sulfisoxazole (SIX). Detailed information on the 20 sulfonamide compounds is presented in Supplementary Table S1.

### 2.2. Instruments and Equipment

QTRAP 5500+ Liquid Chromatography-Mass Spectrometry (LC-MS) System (with Electrospray Ionisation (ESI) source): AB SCIEX, Inc., Marlborough, MA, USA; ME802E Analytical Balance: Mettler-Toledo Instruments Co., Ltd. (Shanghai, China); Ultrapure Water System: Sartorius Scientific Instruments Co., Ltd. (Beijing, China); Handheld Shaker: Juyan Technology Co., Ltd. (Guangdong, China); Constant-Rate Pressure-Injection Extractor: Juyan Technology Co., Ltd. (Taiwan, China).

### 2.3. Solution Preparation and Sample Preparation

A 0.1% aqueous formic acid solution was prepared by accurately pipetting 100 μL of formic acid and diluting to 100 mL with ultrapure water. A 2% acetic acid–acetonitrile solution was prepared by accurately pipetting 2 mL of acetic acid and making it up to 100 mL with acetonitrile. Individual stock solutions of each target compound were prepared by dissolving 10.00 mg of the standard in methanol and making the volume up to 10 mL, resulting in a mass concentration of 1.0 mg/mL; these were transferred to amber standard bottles and stored at −18 °C in the dark. Upon use, an appropriate volume of each individual standard stock solution was accurately pipetted and serially diluted with methanol to prepare a 100 ng/mL mixed standard working solution. Matrix-matched standard working solutions were prepared using blank matrix solutions obtained from sample pretreatment, with concentration levels of 1, 2, 5, 10, 20, 50 and 100 ng/mL.

A 5.00 g portion of homogenized pork was accurately weighed into a 50 mL centrifuge tube. Subsequently, 5.00 g of anhydrous sodium sulfate and one homogenization bead were added, and the mixture was shaken for 5 min. Then, 10 mL of acetonitrile containing 2% acetic acid was added. The sample was thoroughly mixed, shaken for 15 min, and centrifuged at 6000 r/min (4000 rpm) for 5 min. An aliquot of mL of the supernatant was transferred onto a FaVEx-NM cleanup column for purification. Positive pressure was applied to maintain the flow rate at approximately one drop per second, and the entire eluate was collected. The purified solution was then filtered through a 0.22 μm membrane prior to UHPLC-MS/MS analysis. The 5 mL aliquot subjected to cleanup represented one-half of the total 10 mL extract obtained from the 5.00 g pork sample. Quantification was performed using matrix-matched calibration. The final concentration of each analyte in pork was calculated by considering the sample mass, total extraction volume, aliquot volume used for cleanup, and any final concentration or dilution steps during sample preparation. The analyte concentration in pork was calculated according to equation below:

Cs (μg/kg) = (Cf × Vf × Ve)/(m × Va)

where Cs is the concentration of the analyte in the pork sample (μg/kg), Cf is the analyte concentration in the final test solution (ng/mL), Vf is the final volume of the test solution (mL), Ve is the total extraction volume (mL), Va is the volume of extract taken for cleanup (mL), and mL is the sample mass (g). Appropriate unit conversion was applied to express the final result in μg/kg.

### 2.4. Chromatographic and Mass Spectrometric Conditions

#### 2.4.1. Liquid Chromatography Conditions

A Waters ACQUITY UPLC BEH C18 column (2.1 mm × 100 mm, 1.7 μm) was used for the separation of the target compounds. The column temperature was set at 40 °C, the injection volume was 2 μL, and the flow rate was 0.30 mL/min. The mobile phase consisted of a 0.1% formic acid aqueous solution (A) and methanol (B), and separation was performed using gradient elution; the specific gradient elution programme is shown in Table 1.

During the initial stage, a high proportion of the aqueous phase was maintained to facilitate the retention and separation of highly polar analytes. The organic phase proportion was then gradually increased to elute compounds with medium to low polarity, followed by re-equilibration of the column at the end of the run.

#### 2.4.2. Mass Spectrometry Conditions

Mass spectrometry analysis was performed using an electrospray ionisation (ESI) source, with data acquisition conducted in both positive and negative ion modes, and multiple reaction monitoring (MRM) was employed for the qualitative and quantitative analysis of target compounds. The main mass spectrometry parameters are set as follows: ion spray voltage (IS) is 5500 V (positive ion mode) and −4500 V (negative ion mode); curtain gas (CUR) pressure is 35 psi; ion source temperature (TEM) is 500 °C; ion source gas 1 (GS1) pressure is 50 psi; auxiliary heating gas (ion source gas 2, GS2) pressure is 50 psi; collision-activated dissociation gas (CAD) is set to High; resolution is set to Unit; and the MRM detection window is set to 60 s.

Mass spectrometric parameters were optimized by direct infusion of individual standard solutions combined with flow injection analysis. Declustering potential (DP) values were evaluated over an appropriate voltage range at 5 V increments, and the value providing the highest and most stable precursor-ion response was selected. Collision energy (CE) values were subsequently optimized for each precursor-to-product ion transition, also at 5 V increments, to maximize the abundance and stability of the characteristic product ions. For each analyte, one transition was selected for quantification, while at least one additional transition was used for confirmation. The optimized MS/MS parameters are summarized in Table 2.

### 2.5. Data Processing

UHPLC–MS/MS data were processed using SCIEX OS v2.0 software. Target analytes were identified based on retention time, quantifier and qualifier transitions, and ion abundance ratios. The retention time of each analyte in the sample was required to correspond to that of the respective analyte in the matrix-matched standard solution. In addition, both quantifier and qualifier transitions had to be detected, and the ion abundance ratio in the sample had to agree with that in the matrix-matched standard at the same or a comparable concentration level for confirmation of a suspected positive result.

Quantification was performed using the external standard method. Matrix-matched calibration curves were constructed by plotting the mass concentration of each analyte in the matrix-matched standard working solutions against the peak area of the corresponding quantifier transition. The concentration of each analyte in the sample extract was calculated from the calibration curve, and the residue level in the original sample was determined after correction for sample weight, extraction volume, and dilution factor. Results were expressed as μg/kg.

## 3. Results

### 3.1. Optimisation of Mass Spectrometric Conditions

To obtain high sensitivity and stable MS responses, compound-dependent mass spectrometric parameters were optimised for each target analyte. Potential precursor ions were initially inferred from the compound names, molecular formulae, and relative molecular masses. Individual standard solutions were then directly infused into the electrospray ionisation source using a peristaltic pump. Full-scan MS analyses were performed in both positive and negative ionisation modes to determine the molecular ion peaks and select the precursor ions. Ion source-related parameters, including the declustering potential (DP), were subsequently optimised to maximise precursor ion intensity.

Product ion scans were then conducted for the selected precursor ions to obtain characteristic fragment ions by collision-induced dissociation. Two or three fragment ions were selected as monitoring ions according to their response intensity, stability, and specificity. The transition showing the highest and most stable response was used as the quantifier ion pair, while the remaining characteristic transitions were used as qualifier ion pairs. Collision energy (CE) and other compound-dependent parameters were further optimised to establish the final multiple reaction monitoring (MRM) conditions.

Most analytes showed stronger responses in ESI positive mode, providing high sensitivity and favourable signal-to-noise ratios, whereas a few compounds were monitored in negative mode according to their ionisation behaviour. The final MS parameters, including precursor ions, product ions, DP values, and CE values, are summarised in Table 2.

### 3.2. Optimisation of Chromatographic Conditions

To achieve efficient separation of the 20 target analytes and obtain satisfactory peak shapes, the chromatographic conditions were systematically optimised. The initial conditions for positive ionisation mode were established with reference to previously reported methods, and the mobile phase composition and gradient elution programme were further evaluated according to the physicochemical properties of the target compounds.

The results showed that the use of 0.1% (*v*/*v*) formic acid in water as the aqueous phase and methanol as the organic phase provided satisfactory chromatographic responses and relatively symmetrical peak shapes for all 20 analytes. The addition of formic acid to the mobile phase improved the ionisation efficiency of the analytes and enhanced their MS responses in positive ion mode. In addition, it contributed to reduced peak tailing, thereby improving the overall sensitivity of the method. Compared with other mobile phase systems, the formic acid aqueous solution–methanol system showed superior performance in terms of peak shape, response intensity, and separation efficiency.

Moreover, under the optimised gradient elution conditions, analytes with identical precursor ions or common characteristic product ions could be effectively separated, reducing co-elution interference during qualitative confirmation and quantitative determination. This improved the selectivity and accuracy of the method. Therefore, 0.1% formic acid in water and methanol were selected as the final mobile phases, and the optimised gradient elution programme was applied in subsequent analyses.

### 3.3. Optimisation of Sample Preparation Methods

#### 3.3.1. Selection of Extraction Solvent

The selection of an appropriate extraction solvent is critical for efficient release of target analytes from complex matrices, effective protein precipitation, and compatibility with subsequent clean-up and instrumental analysis. Acetonitrile and methanol are commonly used organic solvents for the extraction of veterinary drug residues. Preliminary experiments showed that methanol extraction resulted in extracts containing considerable amounts of proteins and other co-extracted matrix components, leading to turbid sample solutions. Such extracts were unfavourable for subsequent purification and could enhance matrix effects, thereby affecting chromatographic peak shapes and MS responses. Therefore, acetonitrile was selected as the basic extraction solvent, and the effects of different acidified acetonitrile systems on extraction efficiency were further investigated.

Seven extraction solvents were evaluated, including acetonitrile, 0.5% acetic acid in acetonitrile, 1% acetic acid in acetonitrile, 2% acetic acid in acetonitrile, 0.5% formic acid in acetonitrile, 1% formic acid in acetonitrile, and 2% formic acid in acetonitrile. The extraction performance was assessed based on the mean recoveries of the 20 target analytes. The results are presented in Figure 1. As shown in Figure 1, when acetonitrile alone was used as the extraction solvent, the recoveries varied among different classes of analytes, and relatively low recoveries were observed for some compounds. This result suggests that acetonitrile alone had limited extraction efficiency for certain analytes, particularly those with high polarity or strong interactions with matrix components. After acidification of acetonitrile, the recoveries of most target analytes increased markedly. The improved recoveries obtained under acidic conditions may be attributed to enhanced desorption of the analytes from the pork matrix and reduced interactions and endogenous matrix components. Acidified acetonitrile can also promote protein denaturation and precipitation, thereby reducing the co-extraction of proteins and other interfering substances. In addition, since sulfonamides are generally weakly acidic compounds, moderate acidification can suppress their dissociation during extraction and thereby favour their transfer into the organic phase. Differences were also observed among the acidified acetonitrile systems. The acetic acid–acetonitrile systems showed favourable extraction performance for several analytes; however, increasing the acetic acid concentration did not continuously improve the recoveries of all compounds, and a slight decrease was observed for a few analytes. This may be related to reduced analyte stability, increased matrix co-extraction, or decreased electrospray ionization efficiency under relatively strong acidic conditions, which may lead to signal suppression during UHPLC-MS/MS analysis. Therefore, the acid concentration should be carefully optimized to achieve a balance between extraction recovery and instrumental response.

In comparison with the other tested solvent systems, 2% acetic acid in acetonitrile provided higher and more consistent recoveries for most analytes, with relatively small differences among different compound classes. These results indicate that this solvent system offered a good balance between extraction efficiency and method applicability. Considering the mean recoveries of the target analytes, extraction consistency across different compound classes, extract clarity, and compatibility with subsequent purification and UHPLC–MS/MS analysis, 2% acetic acid in acetonitrile was finally selected as the extraction solvent for this method.

#### 3.3.2. Selection of Purification Method

The sample matrix was compositionally complex and contained proteins, lipids, pigments, and other endogenous interferents. Insufficient clean-up may lead to pronounced matrix effects, poor chromatographic peak shapes, and unstable MS responses. Therefore, based on the optimised extraction solvent, three purification strategies commonly used for veterinary drug residue analysis, namely QuEChERS, FaVE-NM clean-up columns, and HLB Pro solid-phase extraction (SPE) cartridges, were compared for the 20 target analytes. Analyte recovery was used as the primary evaluation criterion, and the results are shown in Figure 2.

As shown in Figure 2, the purification strategy had a considerable effect on analyte recoveries. When HLB Pro SPE cartridges were used, relatively low recoveries were obtained for some analytes, and large differences were observed among compounds. This suggests that adsorption loss may occur for certain analytes during SPE clean-up. In particular, highly polar compounds or analytes with strong interactions with the sorbent may be excessively retained or incompletely eluted, resulting in reduced recoveries. In addition, the SPE procedure is relatively laborious and time-consuming, which limits its applicability for rapid screening or high-throughput determination of multi-class veterinary drug residues.

The QuEChERS method is simple and efficient for sample clean-up. However, as shown in Figure 2, several analytes exhibited markedly high recoveries, with some values exceeding 120%. This phenomenon may be associated with incomplete removal of co-extracted matrix components. Residual matrix constituents can induce signal enhancement during electrospray ionisation, leading to apparent over-recovery. For simultaneous determination of multiple classes of veterinary drug residues, such excessive and variable recoveries may compromise quantitative accuracy and method robustness.

In contrast, the FaVE-NM clean-up column provided a more balanced purification performance for the 20 target analytes. After FaVE-NM clean-up, the recoveries of most analytes were within an acceptable range, with relatively small variations among compounds. These results indicate that this approach effectively reduced matrix interference while minimising analyte loss. Compared with SPE, FaVE-NM clean-up was simpler and caused less retention-related loss of target analytes; compared with QuEChERS, it reduced abnormal signal enhancement caused by matrix effects, thereby improving the accuracy and reproducibility of the method.

Considering analyte recovery, consistency among different compounds, matrix clean-up efficiency, operational convenience, and compatibility with UHPLC–MS/MS analysis, the FaVE-NM clean-up column was selected as the final purification method in this study.

### 3.4. Evaluation of Matrix Effects

Matrix effects (MEs) are one of the key factors affecting the quantitative accuracy of UHPLC-MS/MS. In electrospray ionisation (ESI), proteins, lipids, phospholipids, inorganic salts and other co-extracted components in the sample matrix may compete with the target compounds during the ionisation process, thereby enhancing or suppressing the spectral response of the target compounds and consequently affecting the accuracy and reproducibility of the analytical results. Consequently, this study evaluated the matrix effect of 20 target drugs in a pork matrix.

The matrix effect was calculated using the ratio of the peak areas of the target compound in the blank matrix-matched standard solution to those in the pure solvent standard solution, according to the following formula: ME (%) = B/A × 100. Here, A represents the peak area of the target compound in the pure solvent standard solution, and B represents the peak area of the target compound in the blank matrix-matched standard solution. It is generally accepted that when ME falls within the range of 80–120%, the matrix effect is weak; when ME < 80%, this indicates significant matrix suppression; and when ME > 120%, this indicates significant matrix enhancement.

The results are shown in Table 3. The ME values for the 20 target drugs in the pork matrix ranged from 75.40% to 110.99%, which is generally within an acceptable range. With the exception of sulphamethoxypyridazine, the ME values for the remaining compounds all fell within the range of 80–120%, indicating that the optimised sample preparation method was able to effectively remove matrix co-extracted interferents and reduce their impact on the ionisation process of the target compounds, resulting in a generally weak matrix effect. The ME value for sulphamethoxypyrimidine was 75.40%, indicating a certain degree of matrix suppression, suggesting that it may be affected by co-extracted residual components in the pork matrix, leading to a reduced mass spectrometric response. No compounds with an ME > 120% were observed, indicating that this method does not exhibit significant matrix enhancement effects and has good matrix suitability. To further correct for the potential influence of the matrix effect on the quantitative results, this study employed matrix-matched standard curves for quantitative analysis, thereby enhancing the accuracy and reliability of the method.

### 3.5. Methodological Evaluation

#### 3.5.1. Linearity, Limit of Detection and Limit of Quantification

Using pork as the blank matrix, a series of matrix-matched standard working solutions were prepared from the pre-treated blank matrix extract, with mass concentrations of 0, 0.5, 1.0, 5.0, 10.0, 20.0 and 50.0 ng/mL. Following sample preparation as described in Section 2.3, analysis was performed under the UHPLC-MS/MS conditions outlined in Section 2.4. A calibration curve was plotted with the mass concentration of the target compound on the x-axis and the area under the quantitative ion peak on the y-axis, yielding the linear regression equations and correlation coefficients (r) for each compound.

The linear equations, correlation coefficients, limits of detection (LOD) and limits of quantification (LOQ) for the 20 target drugs are shown in Table 3. The results indicate that all target compounds exhibit good linear relationships within the concentration ranges examined, and the correlation coefficients meet the requirements for quantitative analysis. In accordance with the GB/T 33260 [28] standard method, the concentration corresponding to a signal-to-noise ratio (S/N) of approximately 3 was defined as the LOD, and the concentration corresponding to an S/N ratio of approximately 10 was defined as the LOQ. The results showed that the LODs and LOQs for all compounds were low, indicating that the method possesses good sensitivity and can meet the requirements for the trace detection of 20 veterinary drug residues in pork.

#### 3.5.2. Accuracy and Precision

To evaluate the accuracy and precision of the FaVEx one-step purification method for the determination of target veterinary drug residues, blank pork samples confirmed to be free of the target analytes were selected for spiked recovery experiments. Three spiking levels, namely 1, 3, and 5 μg/kg, were assessed. Sample preparation was performed using the FaVEx-NM purification column according to the procedure described in Section 2.3. Six replicate samples were analysed at each spiking level, and the experiment was repeated three times. The mean recoveries and relative standard deviations (RSDs) of each target analyte were calculated, and the results are shown in Table 4.

As shown in Table 4, satisfactory recovery results were obtained for all 20 target veterinary drugs at the three spiked levels. At the 1 μg/kg spiked level, the mean recovery rates for the compounds ranged from 71.78% to 108.85%, with RSDs ranging from 4.22% to 10.91%; at the 3 μg/kg spiking level, the average recovery rates ranged from 72.38% to 112.20%, with RSDs ranging from 4.23% to 10.75%; and at the 5 μg/kg spiking level, the average recovery rates ranged from 70.95% to 109.27%, with RSDs ranging from 2.75% to 9.89%. Overall, the recovery rates for the 20 target compounds at different spiked levels ranged from 70.95% to 112.20%, with RSDs all below 11%, indicating that the method possesses good accuracy and precision.

For most analytes, recoveries were mainly distributed between 80% and 110%, suggesting that the FaVEx-NM purification column effectively removed interfering components from the pork matrix while maintaining satisfactory extraction efficiency for the target compounds. Although the recoveries of a few analytes were slightly lower than 80% or higher than 110% at certain spiking levels, they remained within the generally acceptable range for veterinary drug residue method validation. The low RSDs obtained at all spiking levels further demonstrate the good repeatability of the method and its suitability for quantitative determination of multiple veterinary drug residues in pork samples. In summary, the FaVEx one-step purification method combined with LC–MS/MS provided satisfactory recovery and precision at low, medium, and high spiking levels, and is suitable for the accurate determination of 20 veterinary drug residues in pork.

### 3.6. Analysis of Real Pork Samples

To evaluate the practical applicability of the developed method, 50 real pork samples collected by the Zhoushan Institute for Food and Drug Control were analyzed using the optimized analytical procedure. None of the 20 target sulfonamides were detected above their respective limits of quantification in any of the samples. These results demonstrate that the proposed method is suitable for routine monitoring and safety assessment of sulfonamide residues in pork.

### 3.7. Comparison with Previously Reported Methods

To further evaluate the analytical performance and practical applicability of the proposed method, a narrative comparison was made with representative previously reported approaches for the determination of sulfonamides in pork and other animal derived matrices. Conventional SPE-based methods generally provide effective purification and satisfactory sensitivity; however, they often require multiple conditioning, loading, washing, and elution steps, which may increase solvent consumption, pretreatment time, and sample-handling workload. In comparison, the present method employs a FaVEx filter-based cleanup cartridge, which simplifies the cleanup procedure and improves sample throughput while maintaining satisfactory method performance under the optimized conditions.

QuEChERS-based methods have also been widely used for veterinary drug residue analysis because of their simplicity and broad applicability. Nevertheless, additional dispersive cleanup steps are often required to reduce matrix interference in complex animal-derived samples. The proposed method provides an alternative cleanup strategy with a relatively simple workflow and stable instrumental response for multiple sulfonamides in pork samples.

It should be noted that isotope-labeled internal standards were not used in the present study. Instead, matrix-matched calibration was applied to compensate for matrix-induced signal suppression or enhancement, allowing for reliable quantification under the optimized conditions. However, the absence of isotope-labeled internal standards remains a limitation, as matrix-matched calibration cannot fully correct for extraction losses, injection variability, or long-term instrumental drift. Therefore, the incorporation of suitable isotope-labeled internal standards should be considered in future studies to further improve method robustness and quantitative accuracy.

Overall, based on the present validation results and comparison with representative SPE- and QuEChERS-based approaches, the proposed method provides a simplified pretreatment procedure and satisfactory analytical performance, suggesting its potential applicability for routine monitoring of sulfonamide residues in pork.

## 4. Discussion

This study established and validated a method for the simultaneous determination of 20 veterinary drug residues in pork using FaVEx one-step purification combined with UHPLC–MS/MS. The method involves simple and rapid sample preparation and can effectively remove proteins, lipids, phospholipids, and other co-extracted interferents from the pork matrix, thereby reducing the influence of the complex matrix on the mass spectrometric responses of the target analytes. It is therefore suitable for the rapid screening and quantitative analysis of multiple veterinary drug residues in foods of animal origin.

Method validation results showed that all 20 target compounds exhibited good linearity within the investigated concentration ranges, with correlation coefficients (R^2^) ranging from 0.9922 to 0.9998. The limits of detection and limits of quantification were 0.05–0.74 μg/kg and 0.09–1.48 μg/kg, respectively, demonstrating high sensitivity and meeting the requirements for trace analysis of veterinary drug residues in pork. Evaluation of matrix effects showed that the matrix effect (ME) values of the target compounds in pork ranged from 75.40% to 110.99%. Except for a few compounds showing some degree of matrix suppression, the matrix effects for most analytes were within an acceptable range. Quantification based on matrix-matched calibration effectively compensated for potential bias caused by matrix effects, thereby improving the accuracy of the analytical results.

The spiked recovery experiments further demonstrated the reliability of the developed method. At three spiking levels of 1, 3, and 5 μg/kg, the mean recoveries of the 20 target compounds ranged from 70.95% to 112.20%, with relative standard deviations all below 11%, indicating good accuracy, precision, and reproducibility.

The spiked recovery experiments further demonstrated the reliability of the developed method. At three spiking levels of 1, 3,and 5 μg/kg, the mean recoveries of the 20 target compounds ranged from 70.95% to 112.20%, with relative standard deviations all below 11%, indicating good accuracy, precision, and reproducibility. Since the validation experiments were performed under repeatability conditions and were not repeated on different days, only intra-day repeatability was assessed in this study; inter-day precision was not evaluated.

## 5. Conclusions

In summary, the developed FaVEx one-step purification–UHPLC–MS/MS method combines the advantages of simple operation, high analytical efficiency, high sensitivity, and reliable quantification, and can be applied to the routine monitoring and risk screening of 20 veterinary drug residues in pork. This method also provides a useful technical reference for the rapid determination of multiple classes of veterinary drug residues in complex animal-derived food matrices.

## Figures and Tables

**Figure 1:**
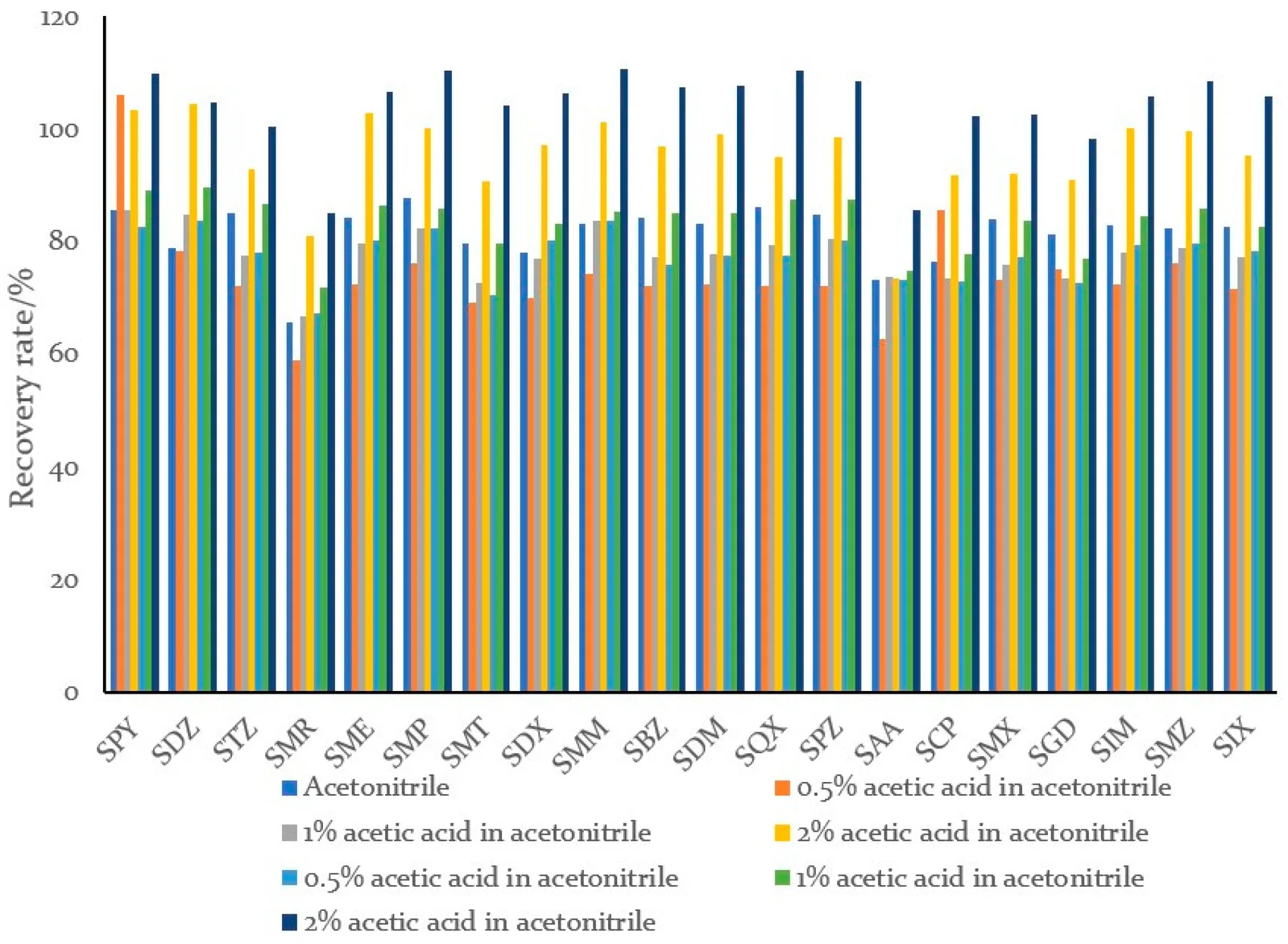
Effects of different extraction solvents on the average recoveries of 20 target drugs (n = 3).

**Figure 2:**
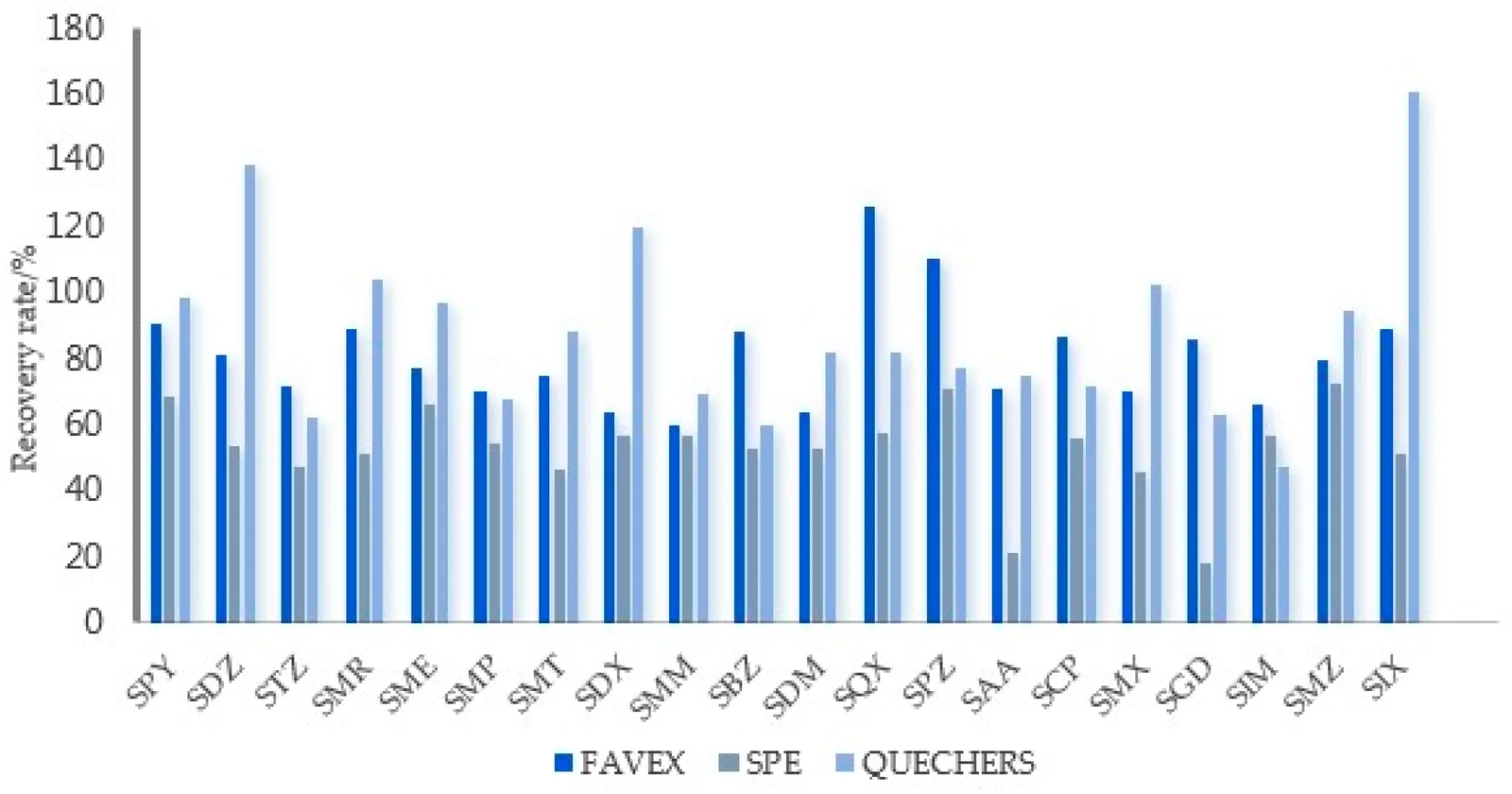
Effects of different clean-up methods on the recoveries of 20 target drugs.

**Table 1:** Mobile phase gradient elution procedure.

Time (min)	Flow Rate (mL/min)	Mobile Phase A (%)	Mobile Phase B (%)
0.0	0.3	98.0	2.0
1.00	0.3	98.0	2.0
13.00	0.3	1.0	99.0
16.00	0.3	1.0	99.0
16.10	0.3	98.0	2.0
20.00	0.3	98.0	2.0

**Table 2:** Compound information and MRM acquisition parameters.

Analyte	Precursor Ion (*m*/*z*)	Quantifier Ion (*m*/*z*)	Qualifier Ion (s) (*m*/*z*)	RT (min)	DP (V)	CE1 (V)	CE2 (V)
Sulfapyridine	250.1	156.1	184.1	4.9	40	23	23
Sulfadiazine	251.1	156	92 92.1	4.29	40	22	38
Sulfathiazole	256	108	92.1	4.69	40	32	37
Sulfamerazine	265.2	156.1	172.1 92.1	5.12	82	25	25 43
Sulfameter	281	156	108.1 92.1	5.98	75	25	35 46
Sulfamethoxypyridazine	281.1	156.1	126.1 108.1	5.62	70	25	27 36
Sulfamethizole	271	156.1	108 92.1	5.72	65	21	36 40
Sulfadoxine	311.11	108.2	92.1	6.65	70	30	70
Sulfamonomethoxine	281.1	156	126.1 108.1 92.1	6.45	75	25	30 36 46
Sulfabenzamide	277.1	156	108	6.98	60	19	32
Sulfadimethoxine	311.1	156.1	218 245.2	7.65	70	28	28 27
Sulfaquinoxaline	301.1	156	108 92.1	7.89	80	24	36 41
Sulfaphenazole	315	156	108 222.1 160.1	7.3	90	27	40 28 31
Sulfacetamide	215	156	108	3.52	52	17	29
Sulfachloropyridazine	285.1	156	108.1	6.23	65	22	37
Sulfamethoxazole	254.1	156	108	6.36	65	22	36
Sulfaguanidine	215	156	92	3.46	80	27	35
Sulfisomidine	279.1	124.1	186.1	5.81	80	30	23
Sulfamethazine/Sulfadimidine	279.1	186.1	156	5.81	60	23	27
Sulfisoxazole	268.1	156.1	113.2 140	6.67	82	22	25 21

**Table 3:** Linearity, LODs, LOQs, and matrix effects of the 20 veterinary drugs.

ID	Linear Equation	R2	LOD/(μg/kg)	LOQ/(μg/kg)	Matrix Effects (ME) (%)
Sulfapyridine	y = 36,615x − 4441.8	0.9997	0.15	0.31	94.81
Sulfadiazine	y = 26,795x − 1208.4	0.9998	0.11	0.22	105.85
Sulfathiazole	y = 13,061x − 1821.8	0.9996	0.18	0.37	102.75
Sulfamerazine	y = 24,139x − 3732.2	0.9995	0.14	0.28	75.40
Sulfameter	y = 50,297x − 3385.4	0.9985	0.2	0.4	106.59
Sulfamethoxypyridazine	y = 47,481x − 8078.2	0.9987	0.34	0.68	94.51
Sulfamethizole	y = 28,172x − 3006	0.9985	0.3	0.6	93.84
Sulfadoxine	y = 32,419x − 5887.8	0.9990	0.3	0.6	93.91
Sulfamonomethoxine	y = 25,436x − 2293.8	0.9999	0.05	0.09	94.06
Sulfabenzamide	y = 59,388x − 8233.5	0.9996	0.14	0.28	85.52
Sulfadimethoxine	y = 10,044x − 257.54	0.9998	0.06	0.12	94.27
Sulfaquinoxaline	y = 44,580x – 11,980	0.9981	0.4	0.8	86.03
Sulfaphenazole	y = 17,578x − 2344.4	0.9997	0.09	0.15	91.13
Sulfacetamide	y = 26,271x − 3183.8	0.9998	0.07	0.14	86.53
Sulfachloropyridazine	y = 28,533x − 3974.6	0.9996	0.14	0.28	94.80
Sulfamethoxazole	y = 27,436x − 2449	0.9994	0.16	0.32	89.40
Sulfaguanidine	y = 5774.6x − 507.93	0.9982	0.4	0.8	91.36
Sulfisomidine	y = 35,514x – 19,497	0.9922	0.74	1.48	93.18
Sulfamethazine/Sulfadimidine	y = 49,722x − 1490.8	0.9993	0.18	0.36	104.36
Sulfisoxazole	y = 23,744x + 2799.7	0.9972	0.36	0.72	110.99

**Table 4:** Spiked recoveries and precision of the 20 veterinary drugs.

ID.	1 (μg/kg)		3 (μg/kg)		5 (μg/kg)	
	Recovery%	RSD%	Recovery%	RSD%	Recovery%	RSD%
Sulfapyridine	82.06	9.49	83.32	8.55	84.54	5.41
Sulfapyridine	73.43	6.47	73.93	7.36	74.29	5.14
Sulfapyridine	88.73	9.35	75.72	7.75	90.48	3.11
Sulfapyridine	88.32	5.62	95.92	4.23	88.90	2.75
Sulfapyridine	87.95	7.07	82.47	8.55	79.45	7.09
Sulfapyridine	86.58	9.85	81.25	9.10	81.58	5.43
Sulfapyridine	71.78	7.58	75.44	10.05	73.27	7.62
Sulfapyridine	88.89	7.66	74.11	7.68	81.85	5.13
Sulfapyridine	87.78	9.85	85.13	8.80	81.52	6.62
Sulfabenzamide	97.34	6.04	90.91	9.24	98.24	5.96
Sulfadimethoxine	89.06	4.22	82.81	9.78	87.74	5.06
Sulfaquinoxaline	97.78	7.58	84.44	8.40	97.06	7.62
Sulfaphenazole	105.52	5.60	76.58	7.74	74.20	7.96
Sulfacetamide	93.52	7.80	112.20	9.95	105.38	9.89
Sulfachloropyridazine	101.45	10.91	91.83	8.64	107.52	7.84
Sulfamethoxazole	108.85	8.81	92.45	5.02	109.27	8.27
Sulfaguanidine	107.57	5.27	102.69	10.75	97.01	9.35
Sulfisomidine	102.85	7.55	98.32	9.74	94.88	8.18
Sulfamethazine/Sulfadimidine	76.65	4.75	72.38	7.02	75.46	8.48
Sulfisoxazole	72.39	8.20	74.90	9.51	70.95	3.93

## Data Availability

The original contributions presented in this study are included in the article/Supplementary Materials. Further inquiries can be directed to the corresponding authors.

## Supplementary Materials

The following supporting information can be downloaded at https://www.mdpi.com/article/10.3390/foods15193465/s1, Table S1: Molecular information and chemical structures of 20 sulfonamide compounds.
