# Supplementary material for: Simultaneous Determination of 20 Sulfonamide Residues in Pork by Filter-Based Solid-Phase Extraction Cleanup and UHPLC–MS/MS
Source: Foods. 2026 Sep 28;15(19):3465. doi: 10.3390/foods15193465 (PMC13664426; doi:10.3390/foods15193465)
Supplement: Supplementary file 1 [file foods-15-03465-s001.zip › foods-4357363-supplementary.pdf]

Table S1. Molecular information and chemical structures of 20 sulfonamide compounds

Compound name	CAS No.	Molecular weight	Molecular Formula	Compound Structure
Sulfapyridine	144-83-2	249.289	C11H11N3O2S	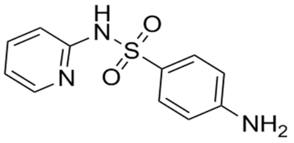
Sulfadiazine	68-35-9	250.28	C10H10N4O2S	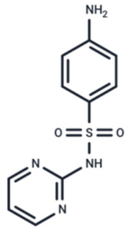
Sulfathiazole	72-14-0	255.32	C9H9N3O2S2	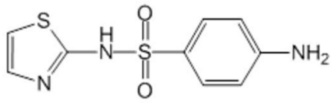
Sulfamerazine	127-79-7	264.30	C11H12N4O2S	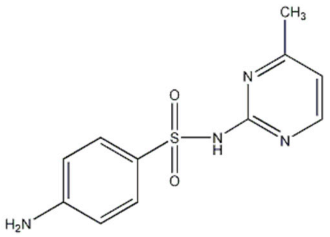
Sulfameter	651-06-9	280.30	$C_{11}H_{12}N_4O_3S$	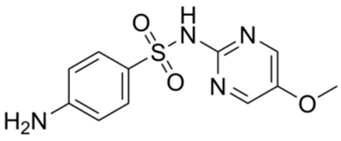

Sulfamethoxypyridazine	80-35-3	280.3	C11H12N4O3S	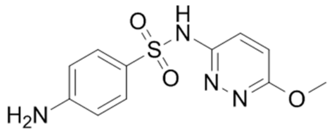
Sulfamethizole	144-82-1	270.33	C9H10N4O2S2	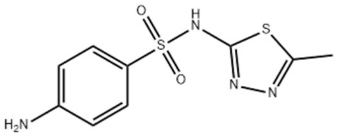
Sulfadoxine	2447-57-6	310.33	C12H10D4N4O4S	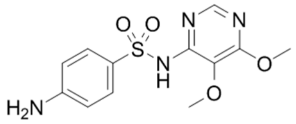
Sulfamonomethoxine	1220-83-3	280.30	$C_{11}H_{12}N_4O_3S$	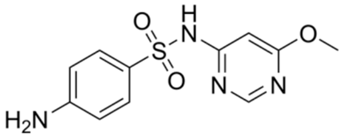
Sulfabenzamide	127-71-9	276.31	C13 H12 N2 O3 S	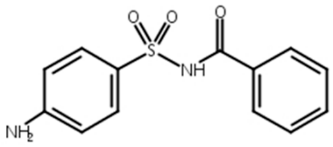
Sulfadimethoxine	122-11-2	310.33	C12H14N4O4S	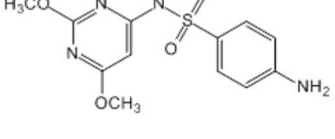
Sulfaquinoxaline	59-40-5	300.34	C14H12N4O2S	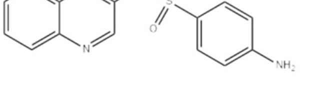

Sulfaphenazole	526-08-9	314.36	C15H14N4O2S	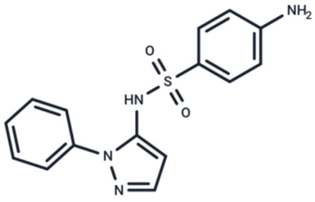
Sulfacetamide	27890-59-1	214.242	C8H10N2O3S	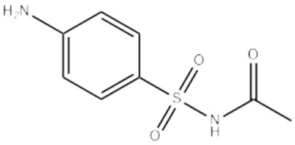
Sulfachloropyridazine	80-32-0	284.72	C10H9ClN4O2S	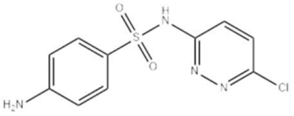
Sulfamethoxazole	723-46-6	257.30	C10H11N3O3S	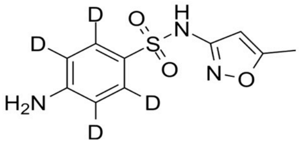
Sulfaguanidine	57-67-0	214.24	C7H10N4O2S	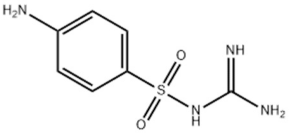
Sulfisomidine	515-64-0	278.33	C12H14N4O2S	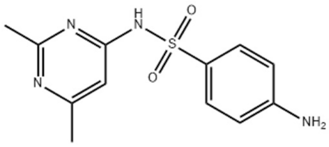
Sulfamethazine /Sulfadimidine	57-68-1	278.33	C12H14N4O2S	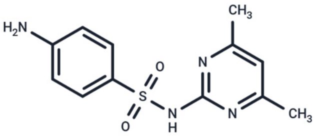
Sulfisoxazole	127-69-5	267.30	C11H13N3O3S	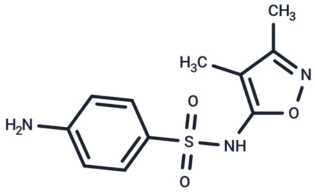
